# Multi-omics analysis elucidates the host-microbiome interplay in severe udder cleft dermatitis lesions in dairy cows

**DOI:** 10.3168/jdsc.2023-0537

**Published:** 2024-03-29

**Authors:** A.S. Vermeersch, F. Van Nieuwerburgh, Y. Gansemans, M. Ali, R. Ducatelle, P. Geldhof, D. Deforce, J. Callens, G. Opsomer

**Affiliations:** 1 Department of Internal Medicine, Reproduction and Population Medicine, Faculty of Veterinary Medicine, Ghent University, 9820 Merelbeke, Belgium; 2 Laboratory of Pharmaceutical Biotechnology, Faculty of Pharmaceutical Sciences, Ghent University, 9000 Ghent, Belgium; 3 Department of Pathobiology, Pharmacology and Zoological Medicine, Faculty of Veterinary Medicine, Ghent University, 9820 Merelbeke, Belgium; 4 Department of Translational Physiology, Infectiology and Public Health, Faculty of Veterinary Medicine, Ghent University, 9820 Merelbeke, Belgium; 5 Dierengezondheidszorg Vlaanderen, 8820 Torhout, Belgium

## Abstract

Udder cleft dermatitis is a skin disease in dairy cattle that is characterized by painful, large open wounds between the udder halves or at the front udder attachment. Its impact on animal welfare and production warrants an in-depth investigation of its pathogenesis. The present study delves into the pathophysiology of severe udder cleft dermatitis, employing a multi-omics approach by integrating transcriptomic and metagenomic data obtained from samples of severe udder cleft dermatitis lesions and healthy udder skin of dairy cattle. All dominant features selected from the virulence factor, taxonomic, and transcriptomic datasets, except for the facultative pathogen *Streptococcus pyogenes*, form a network that could be associated with the healthy udder skin. The severe udder cleft dermatitis–associated *Streptococcus pyogenes* exhibited a negative correlation with these virulence factors and genes, but was not correlated with the other commensal bacteria in the analysis. Examining the different components interacting with each other could advance our understanding of the pathogenesis of severe udder cleft dermatitis.

## Highlights

• Healthy skin-associated genes and virulence factors clustered with commensal microorganisms.
• The selected genes code for components of the skin, membranes, and immune system.
• Dominant healthy skin-associated features such as relB and glyoxalase belong to the virulence factors dataset.
• *Streptococcus pyogenes* was negatively correlated with virulence factors and genes that characterize healthy skin.

## Graphical Abstract

**Figure:**
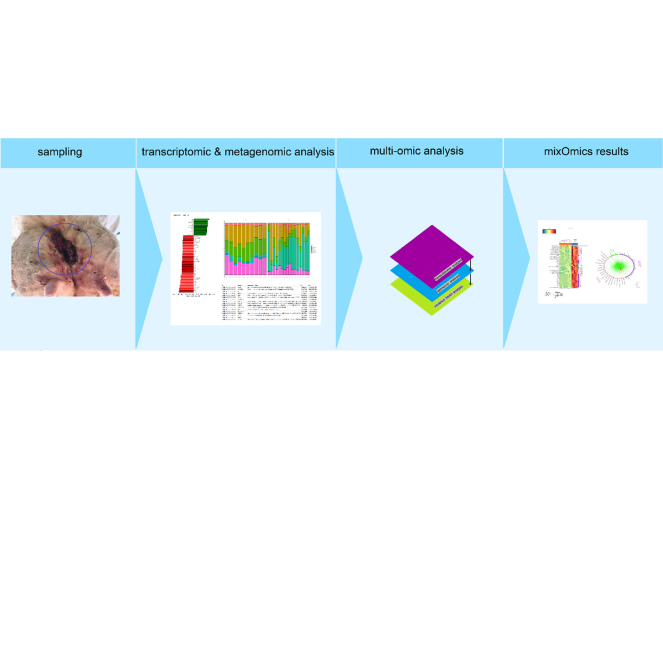
**Summary:** In this study, we analyzed samples from severe udder cleft dermatitis lesions and healthy bovine udder skin through metagenomic and transcriptomic analysis. The resulting omics data were put together in a multi-omics analysis. *Streptococcus pyogenes* was identified as a dominant feature in severe udder cleft dermatitis lesions. Concomitantly, a cluster of commensal bacteria, such as *Bifidobacterium* spp. and *Butyrivibrio* spp., was found together with a set of transcriptomic features and virulence factors consistent with the characteristics of healthy bovine udder skin. The present set of transcriptomic features and virulence factors were negatively correlated with the presence of the facultative pathogen *Streptococcus pyogenes*, highlighting the intricate interplay between microbial composition and the transcriptome in udder skin health.

Intensive livestock farming has given rise to the progressive emergence of intricate, multifactorial diseases such as udder cleft dermatitis (**UCD**), challenging the traditional notions of a singular causal relationship between a pathogen and the host. Udder cleft dermatitis is an inflammatory skin condition affecting dairy cattle (Ekman et al., 2018). Lesions are predominantly located at the front udder attachment or between the udder halves. This disease is characterized by a complex pathogenesis comprising many components such as the microbiome, host response, and environmental influences (Ekman et al., 2018; Vermeersch et al., 2023, 2024). Its specific etiology is still unknown, yet diverse factors contributing to the pathogenesis have been described in the literature. An integrated approach can improve our understanding of the multilayered character of UCD in dairy cattle. The literature on UCD is rather scarce, with the predominant focus on microbiome research, prevalence studies, and risk factor analyses (Ekman et al., 2018, 2020). To date, only one study characterizing the transcriptome (Vermeersch et al., 2023) and another investigating the virulence factors (**VF**) present in the microbiome have been published (Vermeersch et al., 2024). The main objective of the present study was to explore the molecular mechanisms driving the pathophysiology of severe UCD lesions in dairy cattle to obtain novel insights by assessing the integration of omics data on the host response, VF, and dysbiotic microbiome.

The ethical committee of the Faculty of Veterinary Medicine (Ghent University, Belgium) approved the sampling protocol (dossier number 2021–103). All authors followed the ARRIVE guidelines (Percie du Sert et al., 2020). Two dairy farms located in Flanders (Belgium) participated in the transcriptomic (Vermeersch et al., 2023) and metagenomic studies (Vermeersch et al., 2024). According to the classification system published by Persson-Waller et al. (2014) large, exudative open wounds with crusts were categorized as severe UCD lesions. A skilled veterinarian examined the cows for the presence of lesions. The sampled animals were in good health, aside from the severe UCD diagnosis. A detailed description of the materials and methods employed in both studies is available in previously published manuscripts (Vermeersch et al., 2023, 2024). In total, 9,934 genes, 2,646 microbial species, and 133 VF of 15 samples (5 healthy bovine skin and 10 severe UCD samples) were used in the multivariate analysis. Bulk RNA transcriptomics analysis was done to determine the host response, whereas shotgun sequencing, with a concomitant VF analysis by PathoFact (de Nies et al., 2021), led to a taxonomic analysis of the local skin microbiome. Unsupervised partial least squares (**PLS**) analysis with MixOmics in R (v4.2.2) revealed a close-knit relationship (cross-correlations between 88% and 92%) between the metagenomic and transcriptomic datasets of UCD lesions on the first component (Lê Cao et al., 2009; R Core Team, 2021). As a second step, a supervised sparse PLS-discriminant analysis (**sPLS-DA**) with the 3 datasets (transcriptomic, metagenomic, and VF data) as separate blocks was undertaken with a design weight of 0.85. Taking the principle of sparsity into account, a selection of variables was performed, keeping 20 features of the transcriptomic and metagenomic dataset each, and 5 of the VF dataset. As a second step, a supervised multiblock sPLS-DA, also called DIABLO, with the 3 datasets as separate blocks, was undertaken with a design weight of 0.85. Subsequently, a diagnostic analysis was undertaken to assess if the correlations between the components of all 3 datasets had been maximized. According to the DIABLO analysis, correlations between the transcriptomic and VF dataset (97%), transcriptomics and metagenomics dataset (98%), and metagenomics and VF dataset (95%) on the first component were high.

Subsequently, the features contributing to the first component and how they correlate with each other became part of a more in-depth analysis. Figure 1 shows features predominantly belonging to the healthy udder skin group, with the exception of *Streptococcus pyogenes*, a bacterial species with an increased abundance in the UCD group (log fold change [**LFC**] = 2.1; adjusted *P*-value [**PAdj**] = 2.42E-27). Using a high correlation cut-off (r = 0.75), only one negative correlation between *Streptococcus pyogenes* and the transcriptomic and VF datasets was detected (Figure 1). All other variables were positively correlated with each other. *Streptococcus pyogenes* is a known facultative pathogen that can initiate and exacerbate human psoriasis flare-ups (Yousefi et al., 2021). Concomitantly, metagenomic analysis of the complete sample set by Vermeersch et al. (2024) revealed 2 distinct *Streptococcus*-associated VF, speB and thiol-activated cytolysin, to be highly upregulated. The speB promotes skin tissue infection while also possessing pro- and anti-inflammatory capacities (Svensson et al., 2000; Egesten et al., 2009). To the best of our knowledge, the presence of *S. pyogenes* in the microbiome of UCD lesions had not been reported in the literature before. Identifying this potential pathogen as being strongly negatively associated with host genes and VF specifically associated with the healthy udder skin necessitates examining the role of *S. pyogenes* in the pathogenesis of UCD.

**Figure 1:**
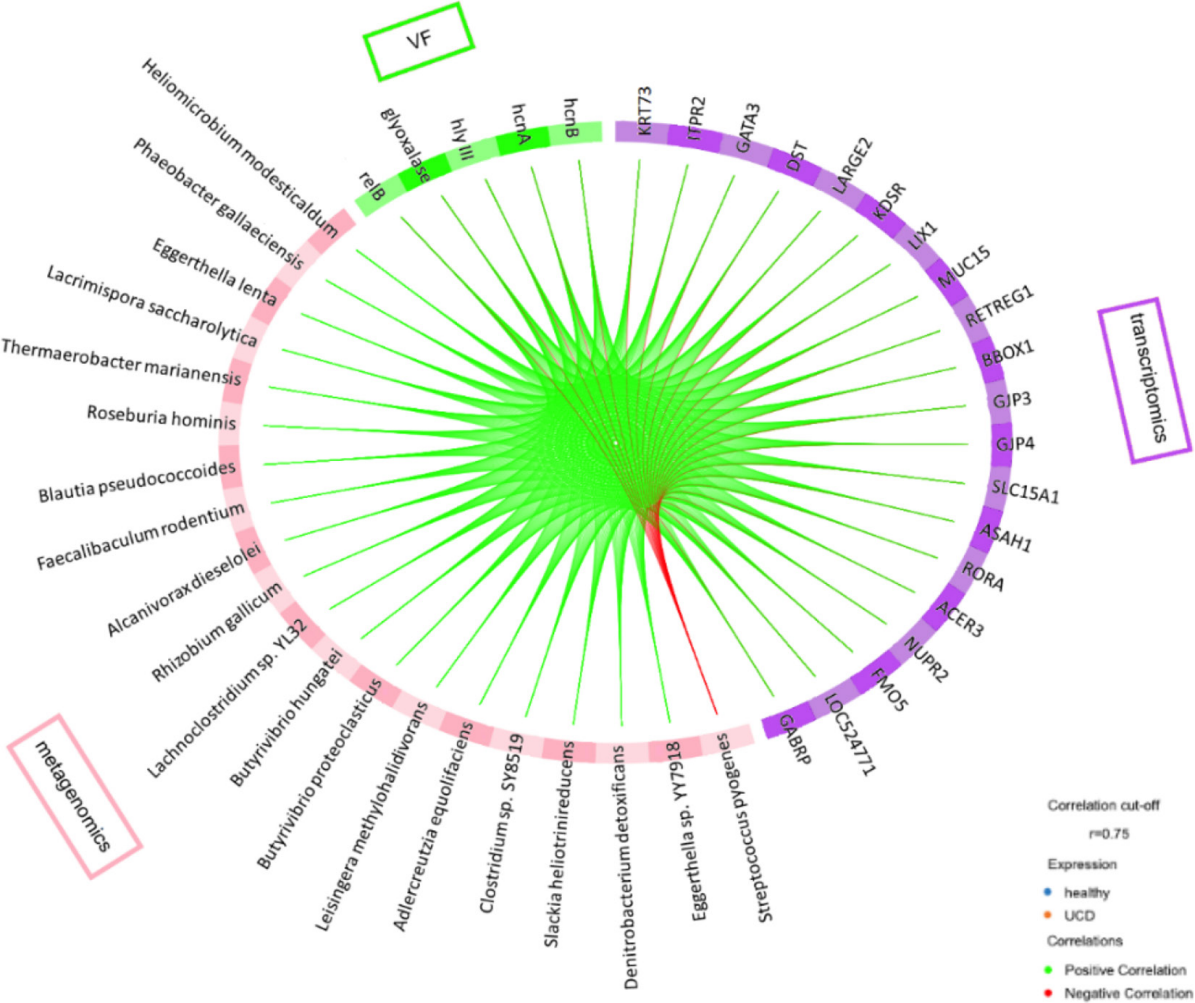
The Circos plot allows us to explore the relationship between the sparse features of the transcriptomic (genes [transcriptomics]) and metagenomic datasets (virulence factors [VF] and microbial species [metagenomics]) associated with severe udder cleft dermatitis, depicted by the colored side quadrants. Strong positive (green) and negative (red) correlations (cut-off = 0.75) between the variables of the datasets are depicted on the plot as lines inside the circle.

Most of the bacterial species surfacing in our multivariate analysis, such as *Butyrivibrio* spp., *Phaeobacter gallaeciensis*, *Adlercreutzia equolifaciens*, and *Faecalibaculum rodentium*, are associated with healthy udder skin. Their decreased abundance in severe UCD lesions not only gives the opportunity for (facultative) anaerobic pathogens to take over, but could also indirectly have a negative impact on the skin barrier and health state of the skin (Sanford and Gallo, 2013; Ekman et al., 2020). *Butyrivibrio* spp. indirectly upregulate the tight junction protein expression through butyrate production (Wang et al., 2012). A decreased abundance of these species might negatively affect tight junction protein expression, with potential implications for the skin barrier. *Adlercreutzia equolifaciens*, a member of the *Eggerthellaceae* family, has shown promising anti-inflammatory properties by inhibiting the NF-κB pathway and reducing the IL-6 expression in mice (Oñate et al., 2023). By reducing retinoic acid production and thereby negatively affecting eosinophil survival, *Faecalibaculum rodentium* has exhibited a positive impact on the epithelial proliferation and turnover in the gut (Cao et al., 2022). In mariculture, researchers are investigating the exploitation of bacterial species such as *Phaeobacter gallaeciensis* as a probiotic to protect fish larvae against bacterial infections such as vibriosis (D'Alvise et al., 2012). The previously mentioned bacteria potentially serve as a protective buffer against the emergence of (facultative) pathogens in a healthy microbiome. Moreover, losing commensal bacteria also diminishes the benefits conferred by this beneficial microbial population (Sanford and Gallo, 2013). Interestingly, among the dominant features is antitoxin relB, which is part of a toxin-antitoxin (**TA**) system in *Escherichia coli* (Gotfredsen and Gerdes, 1998). Additionally, the hcnB and hcnC genes coding for hydrogen cyanide (**HCN**) synthase essentially lead to the production of HCN. A study showed that HCN production by *Pseudomonas aeruginosa* inhibits the growth of the opportunistic pathogen *S. aureus* in in vitro biofilms as well as in a mouse lung model (Létoffé et al., 2022). Further analysis of these VF is imperative to better understand their role in pathogenesis and how they might influence the local microbiome.

Genes associated with several Kyoto Encyclopedia of Genes and Genomes (**KEGG**) metabolic pathways, genetic information processing pathways, and signaling and cellular processes, such as gap junction protein β3 (*GJB3*), gap junction protein β4 (*GJB4*), retinoic acid receptor-related orphan receptor A (*RORA*), GATA binding protein 3 (*GATA3*), and sphingolipid-coding genes, emerged as DIABLO transcriptomic biomarkers. At this point, it is unknown what the downregulation of these specific genes entails for the pathogenesis of UCD. They appear to code for essential components of the skin, membranes, and the immune system (Krivan et al., 1989; Zhou et al., 2004; Common et al., 2005; Ho et al., 2009; Hall et al., 2022). A decrease of those molecules in UCD lesions might compromise the integrity of the skin barrier. Both *GJB3* and *GJB4* code for connexins, a class of gap junction proteins, with mutations leading to an impaired epidermal differentiation (Common et al., 2005). Sphingolipids are membrane constituents and their metabolites are implicated in a lot of orchestrated cellular as well as pathogenic events. They can serve as receptors for natural killer T-cell activation but at the same time can be exploited as a handle for pathogens such as *Pseudomonas aeruginosa* and *Mycoplasma pneumoniae* (Krivan et al., 1989; Zhou et al., 2004). A few other genes (e.g., *RORA* and *GATA3*) play a role in T helper-1, -2, and -17 cell differentiation (Ho et al., 2009; Hall et al., 2022). Interestingly, we found the IL-17 pathway to be one of the major upregulated pathways in severe UCD and digital dermatitis lesions (Vermeersch et al., 2022, 2024). Also, other membrane-associated genes, such as mucin 15 (*MUC15*) and reticulophagy regulator (*RETREG*), appeared in the DIABLO analysis. The hair-specific keratin 73 gene (*KRT73*) emerged as an important variable, belonging to the class of the most downregulated molecules in UCD lesions.

We conclude that the explorative analysis with mixOmics revealed associations between the host response, microbiome, and its associated VF in severe UCD lesions in dairy cattle. The decreased population of commensal bacteria was positively correlated with the downregulation of barrier molecules and genes promoting Th17 cell differentiation. The presence of protective microbes in a commensal, healthy skin microbiome was in line with the results of Vermeersch et al. (2024). An unexpected biomarker for severe UCD surfacing in the multi-omics analysis was *S. pyogenes*, showing negative correlations with all host genes and VF, but not with other microbes in the first component. The generalizability and statistical power of the study could be improved by increasing the sample size while also taking cow- and herd-related factors such as parity, lactation stage, environmental factors, and housing into account. Environment- and host-associated factors could have an impact on the host transcriptome and microbiome. Future research should delve deeper into understanding the mechanisms by which *S. pyogenes* influences the host response and microbial interactions in all UCD stages, shedding light on potential therapeutic interventions and management strategies.

## References

[bib1] Cao Y.G., Bae S., Villarreal J., Moy M., Chun E., Michaud M., Lang J.K., Glickman J.N., Lobel L., Garrett W.S. (2022). *Faecalibaculum rodentium* remodels retinoic acid signaling to govern eosinophil-dependent intestinal epithelial homeostasis. Cell Host Microbe.

[bib2] Common J., O'Toole E., Leigh I., Thomas A., Griffiths W., Venning V., Grabczynska S., Periš Z., Kansky A., Kelsell D. (2005). Clinical and genetic heterogeneity of erythrokeratoderma variabilis. J. Invest. Dermatol..

[bib3] D'Alvise P., Lillebø S., Prol-Garcia M., Wergeland H., Nielsen K., Bergh Ø., Gram L. (2012). *Phaeobacter gallaeciensis* reduces *Vibrio anguillarum* in cultures of microalgae and rotifers, and prevents vibriosis in cod larvae. PLoS One.

[bib4] de Nies L., Lopes S., Busi S.B., Galata V., Heintz-Buschart A., Laczny C.C., May P., Wilmes P. (2021). PathoFact: A pipeline for the prediction of virulence factors and antimicrobial resistance genes in metagenomic data. Microbiome.

[bib5] Egesten A., Olin A., Linge H., Yadav M., Mörgelin M., Karlsson A., Collin M. (2009). SpeB of *Streptococcus pyogenes* differentially modulates antibacterial and receptor activating properties of human chemokines. PLoS One.

[bib6] Ekman L., Bagge E., Nyman A., Persson Waller K., Pringle M., Segerman B. (2020). A shotgun metagenomic investigation of the microbiota of udder cleft dermatitis in comparison to healthy skin in dairy cows. PLoS One.

[bib7] Ekman L., Nyman A., Landin H., Magnusson U., Persson Waller K. (2018). Mild and severe udder cleft dermatitis—Prevalence and risk factors in Swedish dairy herds. J. Dairy Sci..

[bib8] Gotfredsen M., Gerdes K. (1998). The *Escherichia coli* relBE genes belong to a new toxin–antitoxin gene family. Mol. Microbiol..

[bib9] Hall J.A., Pokrovskii M., Kroehling L., Kim B.R., Kim S.Y., Wu L., Lee J.Y., Littman D.R. (2022). Transcription factor RORα enforces stability of the Th17 cell effector program by binding to a *Rorc cis*-regulatory element. Immunity.

[bib10] Ho I.C., Tai T.S., Pai S.Y. (2009). GATA3 and the T-cell lineage: Essential functions before and after T-helper-2-cell differentiation. Nat. Rev. Immunol..

[bib11] Krivan H., Olson L., Barile M., Ginsburg V., Roberts D. (1989). Adhesion of *Mycoplasma pneumoniae* to sulfated glycolipids and inhibition by dextran sulfate. J. Biol. Chem..

[bib12] Lê Cao K.-A., Gonzalez I., Déjean S. (2009). integrOmics: an R package to unravel relationships between two omics data sets. Bioinformatics.

[bib13] Létoffé S., Wu Y., Darch S., Beloin C., Whiteley M., Touqui L., Ghigo J.-M. (2022). *Pseudomonas aeruginosa* production of hydrogen cyanide leads to airborne control of *Staphylococcus aureus* growth in biofilm and in vivo lung environments. MBio.

[bib14] Oñate F.P., Chamignon C., Burz S., Lapaque N., Monnoye M., Philippe C., Bredel M., Chêne L., Farin W., Paillarse J.-M., Boursier J., Ratziu V., Mousset P.-Y., Doré J., Gérard P., Blottière H. (2023). *Adlercreutzia equolifaciens* is an anti-inflammatory commensal bacterium with decreased abundance in gut microbiota of patients with metabolic liver disease. Int. J. Mol. Sci..

[bib15] Percie du Sert N., Hurst V., Ahluwalia A., Alam S., Avey M., Baker M., Browne W., Clark A., Cuthill I., Dirnagl U., Emerson M., Garner P., Holgate S., Howells D., Karp N., Lazic S., Lidster K., MacCallum C., Macleod M., Pearl E., Petersen O., Rawle F., Reynolds P., Rooney K., Sena E., Silberberg S., Steckler T., Wurbel H. (2020). The ARRIVE guidelines 2.0: Updated guidelines for reporting animal research. PLoS Biol..

[bib16] Persson Waller K., Bengtsson M., Nyman A. (2014). Prevalence and risk factors for udder cleft dermatitis in dairy cattle. J. Dairy Sci..

[bib17] R Core Team (2021). R: A language and environment for statistical computing. https://posit.co/products/open-source/rstudio/.

[bib18] Sanford J.A., Gallo R.L. (2013). Functions of the skin microbiota in health and disease. Semin. Immunol..

[bib19] Svensson M.D., Scaramuzzino D.A., Sjöbring U., Olsén A., Frank C., Bessen D.E. (2000). Role for a secreted cysteine proteinase in the establishment of host tissue tropism by group A streptococci. Mol. Microbiol..

[bib20] Vermeersch A.S., Ali M., Gansemans Y., Van Nieuwerburgh F., Ducatelle R., Geldhof P., Deforce D., Callens J., Opsomer G. (2024). An in-depth investigation of the microbiota and its virulence factors associated with severe udder cleft dermatitis lesions. J. Dairy Sci..

[bib21] Vermeersch A.S., Ali M., Gansemans Y., Van Nieuwerburgh F., Geldhof P., Ducatelle R., Deforce D., Callens J., Opsomer G. (2023). Severe udder cleft dermatitis lesion transcriptomics points to an impaired skin barrier, defective wound repair and a dysregulated inflammatory response as key elements in the pathogenesis. PLoS One.

[bib22] Vermeersch A.S., Geldhof P., Ducatelle R., Gansemans Y., Van Nieuwerburgh F., Deforce D., Opsomer G. (2022). Continuous activation of the IL-17F driven inflammatory pathway in acute and chronic digital dermatitis lesions in dairy cattle. Sci. Rep..

[bib23] Wang H.-B., Wang P.-Y., Wang X., Wan Y.-L., Liu Y.-C. (2012). Butyrate enhances intestinal epithelial barrier function via up-regulation of tight junction protein claudin-1 transcription. Dig. Dis. Sci..

[bib24] Yousefi A., Karbalaei M., Keikha M. (2021). Impact of *Streptococcus pyogenes* infection in susceptibility to psoriasis: A systematic review and meta-analysis. World J. Metaanal..

[bib25] Zhou D., Mattner J., Cantu C., Schrantz N., Yin N., Gao Y., Sagiv Y., Hudspeth K., Wu Y.-P., Yamashita T., Teneberg S., Wang D., Proia R.L., Levery S.B., Savage P.B., Teyton L., Bendelac A. (2004). Lysosomal glycosphingolipid recognition by NKT cells. Science.

